# The relationship between coping strategies and infertility self-efficacy with pregnancy outcomes of women undergoing in vitro fertilization: A prospective cohort study

**DOI:** 10.18502/ijrm.v20i7.11556

**Published:** 2022-08-08

**Authors:** Hosna Mirzaasgari, Fereshte Momeni, Abbas Pourshahbaz, Farahnaz Keshavarzi, Masoud Hatami

**Affiliations:** ^1^Department of Clinical Psychology, Faculty of Behavioral Sciences, University of Social Welfare and Rehabilitation Sciences, Tehran, Iran.; ^2^Department of Obstetrics and Gynecology, School of Medicine, Motazedi Infertility Research and Treatment Center, Kermanshah University of Medical Sciences, Kermanshah, Iran.; ^3^Department of Oral and Maxillofacial Medicine, School of Dentistry, Kermanshah University of Medical Sciences, Kermanshah, Iran.

**Keywords:** Coping strategy, Self-efficacy, Infertility, In vitro fertilization, Depression, Anxiety.

## Abstract

**Background:**

Assisted reproductive technology treatments are stressful procedures, but there are individual differences in the emotional response to them. Differences in response to this stress may be related to the outcome of infertility treatment.

**Objective:**

This study aimed to investigate the relationship between coping strategies and infertility self-efficacy with pregnancy outcomes of women undergoing in vitro fertilization/intracytoplasmic sperm injection treatment.

**Materials and Methods:**

This was a prospective cohort study and the 154 infertile women were psychologically evaluated in 2 stages: once before ovarian stimulation and again during embryo transfer. The research measurements used were the Revised COPE, the Depression Anxiety Stress Scale (DASS-21) and the Infertility Self-Efficacy Scale.

**Results:**

There was no significant difference between the group of non-pregnant women and the positive pregnancy group in terms of coping strategies (mental rumination, self-blame, active confronting, goal replacement, avoidance) or self-efficacy in either of the 2 stages of ovarian stimulation and embryo transfer. The Mann-Whitney test did not show any statistical difference between the clinically pregnant women and the only laboratory positive pregnant group. Moreover, the self-blame and mental rumination strategies were positively related with anxiety and depression. Conversely, active confronting, goal replacement, avoidance and self-efficacy were associated with decreased depression, anxiety and stress levels.

**Conclusion:**

It can be concluded that there is no relationship between coping strategies and infertility self-efficacy with in vitro fertilization/intracytoplasmic sperm injection outcomes. Further research is needed to clarify the effects of other psychological factors on the pregnancy outcomes of assisted reproductive treatment.

## 1. Introduction

The number of couples seeking infertility treatment is increasing around the world. The World Health Organization has identified infertility as a major health problem in pregnancy health, which has physical, psychological, and social aspects (1, 2). For most couples, infertility is undeniably a major life crisis and is psychologically stressful. Infertile women can experience severe emotional instability while trying to treat infertility, and worrying about the outcome of treatment can be a major stressor for them. In vitro fertilization (IVF) and intracytoplasmic sperm injection (ICSI) are potentially stressful treatments for participants, which usually last several months, can expose women to greater anxiety, and subsequently, this increased anxiety makes treatment more difficult and can negatively affect the success of treatment (3-5).

Previous research has shown that infertility as a cause of stress can threaten the mental health of infertile individuals and the extent of its impact depends to some extent on cognitive assessment and coping skills (6). Using appropriate coping strategies has a positive impact on the adjustment of stress caused by infertility and stress during treatment (7). Coping strategies are cognitive and behavioral efforts to control and manage stressful life events (8). Some coping strategies may have protective effects, while others may increase the emotional maladjustment of infertile women, which is positively associated with a higher risk of anxiety and depression, which, in turn, may reduce the fertility rate in women undergoing IVF/ICSI treatment (9-11).

In infertile people, self-efficacy is translated into the perception of their ability to use psychological skills to control the emotions associated with infertility. The infertile person with higher self-efficacy will likely have more emotional stability and more insistence on treatment (12, 13). Although IVF therapy is usually stressful for participants, there are individual differences in emotional response to it, and differences in response to this stress may be related to the results of fertility treatment as well as the development of psychiatric problems (6).

Based on the vulnerability-stress model, the role of coping strategies and self-efficacy may be mentioned as vulnerability factors that interact with infertility problems and affect the outcomes of assisted reproductive technology (ART) treatments. Therefore, this study aimed to determine the relationship between coping strategies and infertility self-efficacy (ISE) with the pregnancy outcomes of women undergoing IVF/ICSI treatments.

## 2. Materials and Methods

The present study was a prospective cohort study, which was conducted at the Motazedi Infertility Center affiliated to Kermanshah University of Medical Sciences, Kermanshah, Iran from October 2017 to October 2018.

The sample included 154 women who met the inclusion criteria. According to the statistical methods used and based on the results of previous studies (14), the inclusion criteria were: having a definitive diagnosis of primary infertility, women's age > 37 yr, no previous history of IVF/ICSI, starting initial ART treatment, having at least primary education, and agreeing to participate in the study.

The exclusion criteria were: hormonal diseases such as thyroid hormone disorders, diabetes mellitus, adrenal insufficiency, inappropriate uterine factors (including severe endometriosis), psychiatric disorders or taking psychiatric medicine, male infertility factors (including testicular biopsy), having a history of divorce, and remarriage.

The participant's psychological well-being was evaluated by using demographic questionnaires and the Revised COPE (R-COPE) and Depression Anxiety Stress Scale (DASS-21) 2 times, once at the start of treatment (before the beginning of the ovarian stimulation protocol) and the other before the embryo was transferred into the mother's body. Likewise, the Infertility Self-Efficacy Scale (ISES) was utilized to measure stress and resilience. According to previous studies (15, 16), the level of stress in different stages of ART can vary; for this reason, it is better to evaluate psychological distress in different stages of treatment.

### Measurements

#### The R-COPE

We assessed the amount of coping strategies employed using this 19-item questionnaire (7) which was developed from 2 measurements: 1) the Ways of Coping Questionnaire (WOCQ) with 66 items (8); and 2) a 32-item COPE Revised questionnaire (17) to measure the amount of mental rumination, active confronting, goal replacement, avoidance and self-blame strategies on a 4-point Likert scale (1 = rarely and 4 = frequently). The reliability of the questionnaire was confirmed with a repeatability coefficient of 0.78 for self-blame, 0.88 for mental rumination, 0.87 for active confronting, 0.88 for goal replacement, and 0.94 for avoidance. Moreover, the internal overall reliability of the questionnaire assessed with Cronbach's alpha was equal to 0.78 (7). In the present study, the Cronbach's alpha coefficient was 0.78 for self-blame and 0.87 for mental rumination.

#### ISES

The internal reliability of this 16-item questionnaire was reported to have a Cronbach's alpha of 0.94 (18). The ISES was designed to measure resilience and an individual's self-confidence in coping with infertility diagnosis and treatment. This questionnaire can be used to understand an infertile participant's perception of their ability to use cognitive, emotional, and behavioral skills related to infertility treatment, behavioral-psychological techniques, stress management, use of relaxation techniques, general skills, or self-medication methods. The responses could vary from I am not sure at all (1) to I am sure (5). The score could range from 16-80 with a higher score showing higher self-efficacy. In a study which was performed to evaluate the psychometric properties of the Persian version of the ISES, the Cronbach's alpha was reported to be 0.90 (19). In the present study, the Cronbach's alpha was 0.84.

#### DASS-21

The DASS scale was developed to measure the severity of depression, anxiety, and stress in individuals (20). The Cronbach's alpha coefficient for the depression, anxiety and stress subscales in a normative sample of 717 participants were as follows: 0.81, 0.73 and 0.81, respectively. This scale has 2 major forms: the original 42-item version and a short form (21 items; DASS-21) and in the present study, the DASS-21 was used. The DASS-21 evaluates depression, anxiety, and stress by 7 different terms and has been validated in the Iranian population (21). The DASS-21 is able to detect and screen for symptoms of anxiety, depression and stress over the past wk. It is a self-assessment measure on a 4-point Likert scale. The range of answers varies from “never” to “always." In the present study, the Cronbach's alphas of each subscale were as follows: depression (0.87), anxiety (0.84), and stress (0.86).

As recommended by gynecologists, scientific evidence suggests that there should be at least 3 menstrual periods between microinjection operation and embryo transfer so that the participant's body is neutralized from hormonal medications, which can improve the implantation of the fetus. Participants go to the infertility center at least 3 months after the microinjection to prepare for the embryo transfer stage. Since we did not have access to individuals between embryo transfer and pregnancy test results, in the second phase, the cases were asked to fill out R-COPE and ISES 1 day before the embryo transfer operation.

**Figure 1:**
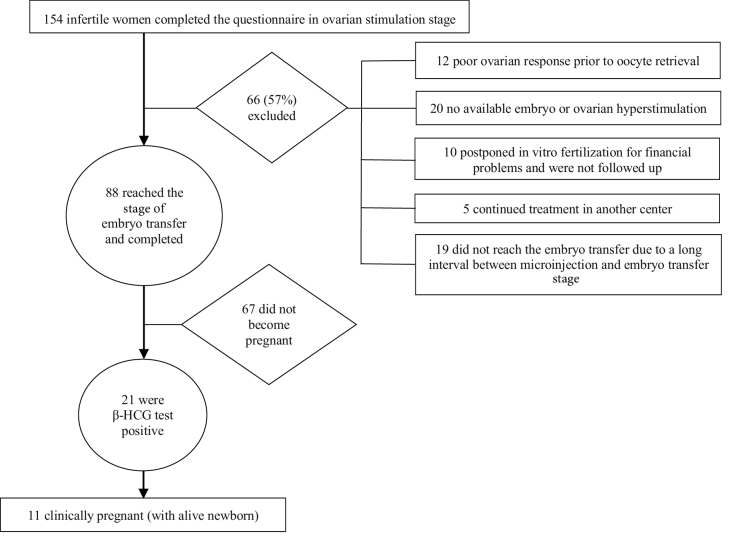
Study method summary.

Among the 154 cases who completed the pre-microinjection questionnaires, only 88 individuals reached the embryo transfer stage and were therefore entered into the statistical analysis (Figure 1). Nineteen out of the 66 excluded participants did not reach the embryo transfer stage because of a long interval between the microinjection and embryo transfer stages. Poor ovarian response led to cancelling the cycles of 12 women before oocyte retrieval. No viable embryos or ovarian hyperstimulation resulted in 20 women having no embryo transfer. 10 cases did not follow the treatments due to financial problems and 5 individuals continued the rest of the treatment cycle in another medical center.

2 wk after the embryo transfer, participants were contacted via phone call to record the positive or negative results of the pregnancy test (β-HCG). Since the criterion for the success of IVF/ICSI treatment was clinical pregnancy in this study, we were not satisfied only with the result of the β-HCG test, but also followed up on the sonography results (fetal heart formation) of the individuals who had a positive β-HCG test after 6-8 wk.

### Ethical considerations

This research was approved on October 18, 2017 by the Ethics Committee of Tehran University of Social Welfare and Rehabilitation Sciences, Tehran, Iran (Code: IR.USWR.REC.1396.180). Before each participant's enrollment, the study protocol was explained completely and informed consent was obtained from each participant.

### Statistical analysis

In this study, the data were analyzed using the Statistical Package for the Social Sciences (SPSS), version 22. Descriptive statistics such as frequency, mean and standard deviation were used to summarize and describe the data. The independent samples *t* test and Chi-square test were used to compare the demographic information of participants. To compare the results of the coping strategies and ISE in women undergoing ART at the 2 stages, the independent *t* test, Mann-Whitney and correlation tests were employed. We used a *t* test to compare the non-pregnant group scores with the positive pregnancy test group scores. Also, we used the Mann-Whitney test to compare the scores of the only positive pregnancy test group with the scores of the clinical pregnancy group.

## 3. Results

The study group included 154 women undergoing IVF/ICSI, of whom only 88 reached the stage of embryo transfer. 67 (76.4%) subjects did not become pregnant and 21 had a positive pregnancy test. 10 (11.4%) women with positive pregnancy tests did not become clinically pregnant and 11 (12.5%) subjects had a clinical pregnancy (live embryo). It is important to note that in some cases, participants neglected to answer 1 or 2 items of the demographic questionnaires. The information on demographic variables as well as fertility variables of all participants is given in table I.

The descriptive statistics of R-COPE and ISES between the non-pregnant, only positive pregnancy test and clinical pregnancy groups in the stages of ovarian stimulation and embryo transfer are shown in table II.

The Chi-square tests showed there was no statistically significant difference in the level of education, socioeconomics or history of previous abortion between the 2 groups (non-pregnant and positive pregnancy test groups). But the 2 groups had a significant difference in theoccupation status (table I), so that 61% of the group with positive pregnancy tests were employed while 11% of the non-pregnant women were employed. Also, based on the independent *t* test results, there were no statistically significant differences in age, duration of infertility, and duration of marriage between the non-pregnant and positive pregnancy test groups. We merged the only positive pregnancy test group with the clinical pregnancy group to increase the number of pregnant women.

Table III shows the results obtained from the independent *t* test for comparing scores of coping strategies and ISE between the groups of the non-pregnant and positive pregnancy test groups in the 2 stages of ovarian stimulation and embryo transfer. According to table III, there was no significant difference between the group of non-pregnant women and the positive pregnancy test group in terms of self-blame, mental rumination, active confronting, goal replacement and avoidance in the stages of ovarian stimulation, and in terms of self-blame, mental rumination, active confronting, goal replacement and avoidance in the embryo transfer stage, as well as in terms of ISE (Table III).

Table IV shows the results from the Mann-Whitney test for comparing the mean scores of the coping strategies and ISE between the group of women with a positive pregnancy test and clinically pregnant women in the 2 stages of ovarian stimulation and embryo transfer. These results indicated that there was no significant difference between the group of the women with a positive pregnancy test and the clinically pregnant women in terms of self-blame, mental rumination, active confronting, goal replacement and avoidance in the stages of ovarian stimulation, and in terms of self-blame, mental rumination, active confronting, goal replacement and avoidance in the embryo transfer stage, as well as in terms of ISE (Table IV).

Furthermore, the results of the correlation test showed a direct and significant relationship between the infertility period and mental rumination and self-blame (Table III).

The coping strategies of self-blame and mental rumination had a significant positive correlation with depression, anxiety and stress but the coping strategies of active confronting, avoidance, goal replacement and ISE had a negative correlation with depression, anxiety and stress (Table V).

**Table 1:** Demographic characteristics and fertility variables for all participants


		**Non-pregnant (n = 67)**	**Positive pregnancy test (n = 21)**	**P-value**
Education*				
	**Middle school and diploma**	18 (26.9) ${}^{*}$	6 (33.3) ${}^{*}$	
	**Diploma and associate**	29 (43.3) ${}^{*}$	8 (44.4) ${}^{*}$	
	**Bachelor and Master's **	20 (31.5) ${}^{*}$	4 (22.2) ${}^{*}$	0.84 ${}^{\text{a}}$
Socioeconomic level*				
	**Low income**	25 (39.6) ${}^{*}$	11 (73.3) ${}^{*}$		
	**Moderate income**	34 (54.0) ${}^{*}$	4 (26.6) ${}^{*}$	
	**High income**	4 (6.4) ${}^{*}$	1 (6.6) ${}^{*}$	0.11 ${}^{\text{a}}$
Occupation status*				
	**Housewife**	44 (89) ${}^{*}$	7 (39) ${}^{*}$		
	**Employed**	5 (11) ${}^{*}$	11 (61) ${}^{*}$	0.001 ${}^{\text{a}}$
History of previous abortion*				
	**Yes**	10 (19.6) ${}^{*}$	5 (29.4) ${}^{*}$	
	**No**	41 (80.4) ${}^{*}$	12 (70.5) ${}^{*}$	0.39 ${}^{\text{a}}$
Age**		31.39 ± 5.05	30.19 ± 4.09		0.32 ${}^{\text{b}}$
Duration of marriage (yr)**		7.50 ± 3.67	7.35 ± 3.31	0.88 ${}^{\text{b}}$
Duration of infertility (yr)**		6.02 ± 3.98	6.15 ± 4.36	0.90 ${}^{\text{b}}$
*Data presented as numbers (Percent). **Data presented as Mean ± Standard deviation. ${}^{\text{a}}$ Chi-square test, ${}^{\text{b}}$ Independent *t* test				

**Table 2:** Descriptive statistics of coping strategies and ISE in the stages of ovarian stimulation and embryo transfer


**Treatment stages**		**Non-pregnant**	**Positive pregnancy test **	**Clinical pregnancy**
**Ovarian stimulation stage**				
	**Self-blame**	6.56 ± 3.5	6.44 ± 3.4	7.63 ± 4.5
	**Mental rumination**	12.29 ± 5.70	12.67 ± 5.02	11.88 ± 6.20
	**Active confronting**	11.50 ± 3.89	10.77 ± 2.64	9.59 ± 2.94
	**Goal replacement**	10.61 ± 2.37	6.67 ± 1.50	5.63 ± 3.06
	**Avoidance**	12.46 ± 4.57	10.89 ± 4.50	9.88 ± 4.22
**Embryo transfer stage**				
	**Self-blame**	5.40 ± 3.36	10.43 ± 5.35	8.14 ± 5.80
	**Mental rumination**	9.96 ± 5.76	11.86 ± 5.80	11.86 ± 5.80
	**Active confronting**	8.35 ± 2.40	7.43 ± 1.87	8.63 ± 2.22
	**Goal replacement**	6.49 ± 2.60	8.00 ± 2.00	6.29 ± 1.27
	**Avoidance**	13.48 ± 2.90	12.57 ± 3.78	13.57 ± 3.70
**ISE**		57.90 ± 10.02	54.67 ± 8.91	55.18 ± 11.67
Data presented as Mean ± Standard deviation. ISE: Infertility self-efficacy				

**Table 3:** Comparison of coping strategies and ISE between the non-pregnant and positive pregnancy test groups in the 2 stages of ovarian stimulation and embryo transfer


**Treatment stages**		**Mean difference (SD)**	**P-value**
**Ovarian stimulation stage**			
	**Self-blame**	-2.43	0.01
	**Mental rumination**	-1.98	0.20
	**Active confronting**	1.98	0.13
	**Goal replacement**	0.55	0.35
	**Avoidance**	0.43	0.69
**Embryo transfer stage**			
	**Self-blame**	-0.75	0.48
	**Mental rumination**	-0.13	0.93
	**Active confronting**	1.98	0.21
	**Goal replacement**	0.73	0.30
	**Avoidance**	1.35	0.24
**ISE**		5.02	0.08
ISE: Infertility self-efficacy, SD: Standard deviation, Data were analyzed by independent *t* test			

**Table 4:** Comparison of coping strategies and ISE between the groups of only positive pregnancy test and clinically pregnant women in the 2 stages of ovarian stimulation and embryo transfer


**Treatment stages**		**Mann-Whitney (Z score)**	**Mean ** ± **SD**	**Median**	**P-value**
**Ovarian stimulation stage**					
	**Self-blame**	-0.90	8.56 ± 3.22	9	0.70
	**Mental rumination**	-0.37	13.88 ± 5.43	15	0.36
	**Active confronting**	-0.69	20.25 ± 4.86	20.5	0.48
	**Goal replacement**	-1.10	6.50 ± 2.44	6	0.29
	**Avoidance**	0.00	11.38 ± 3.18	12	1.00
**Embryo transfer stage**					
	**Self-blame**	-1.18	6.50 ± 3.68	6	0.23
	**Mental rumination**	-0.21	10.50 ± 4.91	9.5	0.83
	**Active confronting**	-1.27	18.63 ± 5.78	20.5	0.20
	**Goal replacement**	-1.37	5.94 ± 2.08	6	0.25
	**Avoidance**	-0.37	11.13 ± 3.09	11	0.70
**ISE**		-0.95	53.19 ± 9.50	54	0.33
Data were analyzed by Mann-Whitney test. ISE: Infertility self-efficacy, SD: Standard deviation					

**Table 5:** Correlation between coping strategies and ISE with depression, anxiety and stress


		**Depression**	**Anxiety**	**Stress**
**ISE**		r = -0.64, p < 0.01	r = -0.59, p < 0.01	r = -0.66, p < 0.01
**Ovarian stimulation stage**				
	**Self-blame**	r = 0.52, p < 0.01	r = 0.52, p < 0.01	r = 0.53, p < 0.01
	**Rumination**	r = 0.44, p < 0.01	r = 0.40, p < 0.01	r = 0.46, p < 0.01
	**Active confronting**	r = -0.33, p < 0.01	r = -0.30, p = 0.02	r = -0.30, p = 0.01
	**Avoidance**	r = -0.27, p = 0.01	r = -0.33, p < 0.01	r = -0.30, p = 0.01
	**Goal replacement**	r = -0.30, p = 0.01	r = -0.23, p = 0.04	r = -0.20, p = 0.07
**Embryo transfer stage**				
	**Self-blame**	r = 0.40, p < 0.01	r = 0.19, p = 0.12	r = 0.37, p < 0.01
	**Rumination**	r = 0.32, p = 0.01	r = 0.16, p = 0.19	r = 0.03, p = 0.01
	**Active confronting**	r = -0.47, p < 0.01	r = -0.48, p < 0.01	r = -0.44, p < 0.01
	**Avoidance**	r = -0.45, p < 0.01	r = -0.37, p < 0.01	r = -0.33, p < 0.01
	**Goal replacement**	r = -0.47, p < 0.01	r = -0.40, p < 0.01	r = -0.40, p < 0.01
ISE: Infertility self-efficacy, r: Pearson correlation coefficient, p = P-value				

## 4. Discussion

The results of this study showed that 24.0% of the participants had a positive pregnancy test result (β test). However, only 12.5% of them were clinically pregnant and gave birth to a live fetus. Most studies consider a positive pregnancy test (β test) as a successful result of IVF/ICSI, but it does not appear to be a good indicator of the success of ART. Thus, in the present study, the criterion for the success of treatment was clinical pregnancy (live fetal heart formation in the 6 ${}^{\text{th}}$ wk at ultrasonography) and therefore it is important to be compared to other similar studies.

The results of this study showed that there was no statistically significant relationship between coping strategies and ISE with women's IVF/ICSI outcomes. These findings may be due to the low sample size in the 2 groups of the only positive β test pregnancy and clinically pregnant women.

According to the stress–vulnerability model, people are likely to respond differently to stressful life events than others, either because of biological predispositions or because of initial sensitivities (22). The use of appropriate coping strategies has a positive effect on the regulation of psychological distress caused by infertility and stress during treatment (23, 24).

The present study confirmed the positive relationship between the self-blame and mental rumination strategies with anxiety and depression, similarly to other studies (Table V) (10, 25, 26). Also, this study demonstrated that the active confronting coping strategy, unlike rumination and self-blame, was related to decreased depression and anxiety levels. The active confronting strategy is recognized as an adaptive strategy in experiencing crises, while in Kazemi et al.'s study, this strategy was associated with higher depression and anxiety in infertile males (27). Also, this study noted that the avoidance coping strategy was negatively related with the depression and anxiety scores of the participants. This finding is in accordance with the results of the Driscoll et al. study (28). In accordance with some other studies, our research showed that the goal replacement strategy can be associated with reduced depression and anxiety in infertile couples (29, 30).

It should be noted that the behavioral and adaptive coping strategies are situation specific and dependent on the interplay between the individual and situations (31). The effects of coping strategies as maladaptive or adaptive strategies on mental health is controversial. These discrepancies are common due to the diversity of situations and personalities that show the complex nature of the relationship between coping strategies and psychological states.

Furthermore, the results showed that there was no statistically significant relationship between the ISE scores and women's pregnancy outcomes under ART. This result is inconsistent with the results of the Turner et al. study (16). They revealed that high scores of ISE in the stage of oocyte retrieval were associated with higher pregnancy rates.

Bandura's theory of self-efficacy shows that people with high self-efficacy are confident, resilient, and find solutions to problems by mastering the environment in which they put themselves at risk. In contrast, people with low self-efficacy underestimate their power and ability, have a poor commitment to achieving goals, and show negative emotional reactions such as anxiety and fear (32). Self-efficacy plays a major role in the following of health behaviors such as therapeutic follow-up and safety behaviors. High self-efficacy not only changes health behaviors but also creates more motivation to strive and persevere when faced with obstacles. Self-efficacy beliefs reduce the influence of stressful situations and can act as a mediator between stress and negative emotions (33).

Among the limitations of the study were the relatively large drop-off in subjects and the low sample size, especially in the case of pregnant women, which made it impossible to compare the groups with the same sample sizes. Additionally, the assessments made in this study were based on self-report scales, so some people may have tried to show themselves as better or worse.

Due to the limitations of the research, it is suggested that future research is conducted with a much higher sample size and in different areas. Also, it is recommended to evaluate the participants' relatives and other tools to more accurately assess the individuals.

## 5. Conclusion

The results of this study showed that there was no relationship between coping strategies and ISE with the pregnancy outcomes of IVF/ICSI treatment. In addition, recent studies have shown that the effects of psychological distress on IVF treatment outcomes are not yet clear, and women's emotional distress does not appear to determine the success of treatment through ART. It can be concluded that the relationship between psychological factors and IVF outcomes is more complex than is usually thought.

##  Conflict of Interest

The authors declare that there is no conflict of interest.
